# Estimating the Risk of Poverty Transitions at Old Age in the United States: A Survival Analysis Focused on the Hispanic Population

**DOI:** 10.1093/geroni/igaa057.320

**Published:** 2020-12-16

**Authors:** Antonia Diaz-Valdes Iriarte, Fidel Bennett Ramos

**Affiliations:** 1 Universidad Mayor, La Reina, Santiago, Region Metropolitana, Chile; 2 Universidad de Chile, Santiago, Chile

## Abstract

Many Americans work well past the Social Security full retirement age. Moreover, the high labor participation rate of aged workers does not translate into better living conditions and decreased poverty at old age. According to the OECD’s relative poverty threshold, the poverty rate at old age is 23.1% (14 points higher than the absolute measure), showing important increases as age progresses. In the context of financial pressure faced by SS, understanding the factors that could help people ensure themselves against poverty is crucial at old age, especially for groups such as Hispanics. Hispanics tend to rely mostly on SS, and tend to retire earlier than non-Hispanics due to limited working opportunities. Using data drawn from the Health and Retirement Study (2008-2016), cox survival models, were conducted to estimate the risk of transitioning into poverty during retirement on determinants of long-term accumulation of disadvantages over the life course (i.e.: wealth accumulation, medical care access, unemployment history). Results suggest that being foreign born and Hispanic are strongly associated with an increase on the probability of experiencing poverty at old age, conditional on health, education and employment status. Furthermore, delaying claims of SS by one year reduces the probability of falling into poverty by 3%, supporting that socioeconomic conditions faced by older adults, especially, the interactions between such factors and ethnicity are key in explaining poverty at old age. This would help policy makers to implement policies could be targeting those factors earlier on, beneficiating the individuals and society as a whole

